# Human Urinary kallidinogenase promotes good recovery in ischemic stroke patients with level 3 hypertension

**DOI:** 10.1002/brb3.752

**Published:** 2017-06-21

**Authors:** Danhong Wu, Yi Lyu, Ping Zhong, Fengdi Liu, Xueyuan Liu

**Affiliations:** ^1^ Department of Neurology Shanghai Fifth People's Hospital Fudan University Shanghai China; ^2^ Department of Medical Affairs Techpool Bio‐pharma Co.; Ltd. Guangzhou China; ^3^ Department of Neurology Shanghai TCM Integrated Hospital Affiliated Shanghai University of Chinese Medicine Shanghai China; ^4^ Department of Neurology Shanghai Tenth People's Hospital of Tongji University Shanghai China

**Keywords:** acute ischemic stroke, human urinary kallidinogenase, hypertension

## Abstract

**Aim:**

To evaluate the clinical efficacy of Human Urinary kallidinogenase (HUK) in the treatment of acute ischemic stroke (AIS) patients with level 3 hypertension.

**Methods:**

In this retrospective study, from January 2015 to June 2016, 150 consecutive AIS patients were registered in our database. Among them, 47 with level 3 hypertension received either HUK treatment (HUK group, 22 cases) or basic treatment (control group, 25 cases). Basic treatment was administrated on all patients. 0.15 PNA unit of HUK injection plus 100 ml saline in intravenous infusion was performed in the HUK group, with once a day for 14 consecutive days. The modified Rankin Scale (mRS) scores in two groups were analyzed 3 months after the treatment.

**Results:**

No difference was found in the NIHSS scores, age, gender, and comorbidities between two groups before treatment (*p *>* *.05). While after treatment, 3‐month mRS score was significantly lower in the HUK group (2.1 ± 1.4 vs. 3.1 ± 1.3, *p *=* *.012) and good recovery rate (3‐month mRS score ≤2) in the HUK group was significantly higher than that in the control group (*p *<* *.05).

**Conclusion:**

HUK is able to promote long‐term recovery for AIS patients with level 3 hypertension remarkably.

## 1 INTRODUCTION

Stroke is the leading cause of death and disability in China (Bazzano et al., 2007) and the second most common cause of death worldwide after coronary heart disease (Reed, 1990). The epidemiological data show that from 1984 to 2004 the incidence of acute ischemic stroke (AIS) as the most common subtype, representing about 80% of all strokes, has increased by 8.7% per year in China (Jia, Liu, & Wang, 2010). Survivors often have severely diminished quality of life and require long‐term care. In the past 50 years, several important risk factors for AIS have been identified, including hypertension, diabetes, smoking, and dyslipidemia (Sever et al., 2003; Wong et al., 2001). However, blood pressure control in acute phase of AIS has been challenging because it can reduce brain perfusion and aggravate infarct volume.

The kallikrein–kinin system consisting of kinins, kallikreins, and kininogens have been shown to protect against ischemic stroke in patients (Zhang et al., 2012) and animal studies (Chen et al., 2010). Kallikrein, a glycoprotein of the serine proteinase superfamily, can cleave a kininogen substrate to produce the potent vasodilator kinin peptide. Then, kinin mediates a complex series of physiological actions through its receptor signaling pathway (Chao & Chao, 2006; Emanuelia & Madeddu, 2003). Recently, kallikrein has been reported to have a number of functions, including selectively dilating arterioles in the ischemic area, enhancing angiogenesis and neurogenesis, increasing regional cerebral blood flow, inhibiting apoptosis and inflammation, promoting glial cell migration, and improving neurological deficits after AIS (Ling et al., 2008; Lu et al., 2008; Nagano, Suzuki, Hayashi, & Asano, 1992; Stone et al., 2009; Xia et al., 2006). As kallikrein can regulate the dilation of arterioles, whether Human Urinary kallidinogenase (HUK), a commercially available kallikrein–kinin system regulating medicine, is efficient in AIS patient with hypertension has not been reported yet. Recent studies have revealed that elevated blood pressure at the early stage is associated with unfavorable outcome in AIS patients (Tien, Chang, Lee, Liaw, & Chen, 2016). Therefore, we conducted a retrospective, registration‐based study to assess the effects of HUK on prognosis in AIS patients with level 3 hypertension.

## 2 METHODS

### 2.1 Patients

From January 2015 to June 2016, 150 consecutive AIS patients, who were admitted in the neurology department of our hospital, were registered in our database. Among them, 47 patients with level 3 hypertension were taken as study subjects. They were in accordance with the diagnostic criteria of cerebral infarction approved by the fourth national cerebrovascular academic conference (1995) (Chinese Neuroscience Society, Chinese Neurosurgical Society, 1996), and confirmed by head CT or MRI. Inclusion criteria for cases: (1) ages ranging from 18 to 90 years; (2) patients with the first onset; (3) onset time less than 48 hr; (4) patients without bleeding disorder or bleeding trends in latest 1 month; (5) patients without incomplete hepatic and renal function; and (6) patients without the medical history of peptic ulcer, hemorrhagic stroke, brain tumor, and brain trauma.

### 2.2 Therapeutic methods

Basic treatment was performed among patients in both two groups according to disease condition, with antiplatelet therapy, statins preparation, neuroprotective agents, dehydrating agents, blood pressure, and blood glucose controlling agents. On that basis, 0.15 PNA unit of HUK injection (Trade name: Kailikang, Guangdong Techpool Bio‐Pharma Co., Ltd. With approved medicine of H20052065) plus100 mL saline in intravenous infusion was taken in the HUK group, with once a day for 14 consecutive days. In the HUK group, during 24 hr before medication and in the treatment period, angiotensin‐converting enzyme inhibitor, steroid drugs, and other therapeutic drugs on cerebral infarction were forbidden.

### 2.3 Study design

This was a single‐center, registry‐based, retrospective study. Patients’ data were obtained from our database system, and patients were divided into two groups according to the treatment they received: 22 cases in the HUK group and 25 cases in the control group. Baseline characteristics included gender, age, comorbidities, and National Institute of Health stroke scale (NIHSS) score before treatment. mRS scores at 3 months were obtained by telephone follow‐up. Patients’ baseline characteristics, 3‐month mRS scores, 3‐month good recovery rate (3‐month mRS score ≤2), and adverse consequences were compared.

### 2.4 Statistical analysis

Categorical variables were reported as number or percentage; continuous variables fitting the normal distribution were expressed as mean ± standard deviation (SD). Patients’ baseline characteristics were compared by the Chi‐squared test or Fisher's exact test as appropriate for categorical variables, and Student's *t* test for continuous variables. *p* values were two tailed and considered statistically significant if <.05. Data analyses were performed using IBM SPSS Statistics v.19 (SPSS Inc., Chicago, IL, USA).

## 3 RESULTS

### 3.1 Baseline characteristics of all the patients

There were 11 males and 11 females in the HUK group, with an average age at 71.7 ± 11.3 years old. Among them, 13 patients had diabetes mellitus and most of them (92.3%) controlled their blood glucose well. The NIHSS score before treatment in the HUK group was 3.7 ± 1.9. In the control group there were 15 males and 10 females with an average age at 72.0 ± 9.4 years old. Eight cases together with diabetes mellitus and the NIHSS score before treatment was 4.3 ± 1.9. No statistically significant difference in baseline characteristics was found between the two groups (Table 1, *p* > .05).

**Table 1:** Basic characteristics of all level 3 hypertension patients

	HUK group (*n* = 22)	Control group (*n* = 25)	*p* value
Age (year, $\overset{¯}{x}$±s)	71.7 ± 11.3	72.0 ± 9.4	.928
Gender (Male) [Case (%)]	11 (50.0%)	15 (60.0%)	.564
Smoking [Case (%)]	3 (13.6%)	7 (28.0%)	.297
Diabetes [Case (%)]	13 (59.1%)	8 (32.0%)	.082
Good control of blood glucose [Case (%)]	12/13 (92.3%)	7/8 (87.5%)	1.000
Hyperlipoidemia [Case (%)]	12 (54.5%)	14 (56.0%)	.920
NIHSS score before treatment ($\overset{¯}{x}$±s)	3.7 ± 1.9	4.3 ± 1.9	.288

### 3.2 Efficacy and safety of HUK

Three‐month mRS scores of the HUK group and the control group were 2.1 ± 1.4 and 3.1 ± 1.3, respectively (*p *=* *.012). Eleven patients in the HUK group (50.0%) and five patients in the control group (20.0%) got good recovery (3‐month mRS score ≤2, Table 2, *p* = .030). No adverse consequence was reported in the HUK group.

**Table 2:** Outcomes of all level 3 hypertension patients

	HUK group (n = 22)	Control group (n = 25)	*p* value
3‐month mRS score ($\overset{¯}{x}$ ± s)	2.1 ± 1.4	3.1 ± 1.3	.012
Good recovery rate [3‐month mRS score ≤ 2, case (%)]	11 (50.0%)	5 (20.0%)	.030

## 4 DISCUSSION

HUK is a state category I new drug approved by China's State Food and Drug Administration (SFDA), and it is widely used for AIS in China. Clinical results have shown that HUK effectively and safely improves neurological deficits induced by AIS (Ding et al., 2007). A systematic review in 2010 on the efficacy and safety of HUK in stroke studied 2,433 patients (24 trials) and demonstrated that 2117 patients (22 trials) benefited from HUK treatment, eliciting 87% efficacy rate (Zhang et al., 2012). However, the authors failed to perform subgroup analysis because of insufficient available data. So it is not yet clear whether the routine use of HUK would be effective and safe enough in AIS patients with hypertension. In this study, HUK promoted favorable recovery in level 3 hypertension AIS patients, suggesting that HUK had the potential of regulating blood pressure in AIS patients.

Pulse pressure is a strong cardiovascular diseases’ risk factor. According to a systematic review, 10 mmHg increase in pulse pressure was associated with 1.046‐fold increased risk of stroke occurrence, including systolic blood pressure (pooled HR 1.053, 95% CI: 1.033–1.073, *p *<* *.001) and diastolic blood pressure (pooled HR 1.056, 95 % CI: 1.038–1.074, *p* < .001) (Liu et al., 2016). Moreover, Tziomalos et al. (2015) reported that elevated diastolic but not systolic blood pressure increased mortality risk in hypertensive patients with AIS, but no such relation was found in normotensive AIS patients.

Experimental and clinical evidence implicated an imbalance between endogenous vasoconstrictor and vasodilator systems in the development and maintenance of hypertension (Madeddu, Emanueli, & El‐Dahr, 2007). Tissue kallikrein levels were reduced in humans and in animal models with hypertension. Transgenic mice or rats overexpressing human tissue kallikrein or kinin B2 receptor were permanently hypotensive (Chao, Bledsoe, Yin, & Chao, 2006). Kinins, including bradykinin and lys‐bradykinin, are endogenous vasodilators and natriuretic peptides known best for their ability to antagonize angiotensin‐induced vasoconstriction and sodium retention. Moreover, kallikrein gene delivery or kallikrein protein infusion can directly improve cardiac, renal, and neurological function through selectively dilating arterioles, thus without blood pressure reduction (Chao et al., 2006).

Researches showed that the B1 kinin receptor contributed to vascular hypertrophy in angiotensin‐II‐induced hypertension, through a mechanism involving reactive oxygen species generation and extracellular signal‐regulated kinase (ERK1/2) activation (Ceravolo et al., 2014). In myocardial infarction produced by ischemia/reperfusion, kinins help reduce infarct size following preconditioning or treatment with angiotensin‐converting enzyme (ACE) inhibitors (Rhaleb, Yang, & Carretero, 2011). Kinins act via nitric oxide (NO) contribute to the vascular protective effect of ACE inhibitors during neointima formation, and kallikrein/kinin leads to increased levels and Akt activation, and reduces reactive oxygen species formation, TGF‐beta1 expression, MAPK, and nuclear factor‐kappa B activation (Chao & Chao, 2005).

HUK can activate kallikrein–kinin system (Sahan et al., 2013), transfer kininogen hydrolysis into kinin and kallidin and combine with BI receptor produced under induction of ischemic brain tissue to release NO and relax vascular smooth muscle (Ariturk et al., 2012; Perilli et al., 2012). So it can expand blood vessels in the ischemic area, improve cerebral blood supply of penumbra, and restore the neurological deficit as soon as possible. Furthermore, kinins could induce the expression of vascular endothelial growth factor (VEGF) and vascular endothelial growth factor receptor 2 (VEGFR2), which then transactivates endothelial NO synthase and promotes new blood vessels formation (Ke & Jing, 2016; Li et al., 2008).

In this study, there is no side effect of hypotension, the most common adverse event of HUK, on the patients using HUK treatment, which provides the evidence on the safety of HUK in the application of AIS patients with level 3 hypertension.

Taken together, this study at first confirmed that HUK could improve favorable recovery in level 3 hypertension AIS patients with great safety because of its property of selectively dilating arterioles in the ischemic area and enhancing angiogenesis. Due to the limitation of the small sample size and short follow‐up period, further investigation is needed in future.

## 5 CONCLUSION

In conclusion, HUK successfully promoted good recovery in AIS patients with level 3 hypertension. Therapeutic strategy of the supplement of kallikreins to increase cerebral blood flow and modulating vascular protection might prevent the development and progression of ischemic cerebral injury.

## CONFLICT OF INTEREST

None declared.
